# Effects of Sub-Acute Manganese Exposure on Thyroid Hormone and Glutamine (Gln)/Glutamate (Glu)-γ- Aminobutyric Acid (GABA) Cycle in Serum of Rats

**DOI:** 10.3390/ijerph16122157

**Published:** 2019-06-18

**Authors:** Chao-Yan Ou, Yong-Hua He, Yi Sun, Lin Yang, Wen-Xiang Shi, Shao-Jun Li

**Affiliations:** 1 Department of Toxicology, School of Public Health, Guilin Medical University, Guilin 541004, China; ouchaoyan@glmc.edu.cn (C.-Y.O.); hyhup@shmu.edu.cn (Y.-H.H.); sunyi@glmc.edu.cn (Y.S.); yanglin@glmc.edu.cn (L.Y.); shiwenxiang@glmc.edu.cn (W.-X.S.); 2 Department of Toxicology, School of Public Health, Guangxi Medical University, Nanning 530021, China

**Keywords:** manganese, thyroid hormone, glutamine (Gln)/glutamate (Glu)–γ-aminobutyric acid (GABA) cycle, serum

## Abstract

Excessive manganese (Mn) exposure may adversely affect the central nervous system, and cause an extrapyramidal disorder known as manganism. The glutamine (Gln)/glutamate (Glu)–γ-aminobutyric acid (GABA) cycle and thyroid hormone system may be involved in Mn-induced neurotoxicity. However, the effect of Mn on the Gln/Glu–GABA cycle in the serum has not been reported. Herein, the present study aimed to investigate the effects of sub-acute Mn exposure on the Gln/Glu–GABA cycle and thyroid hormones levels in the serum of rats, as well as their relationship. The results showed that sub-acute Mn exposure increased serum Mn levels with a correlation coefficient of 0.733. Furthermore, interruption of the Glu/Gln–GABA cycle in serum was found in Mn-exposed rats, as well as thyroid hormone disorder in the serum via increasing serum Glu levels, and decreasing serum Gln, GABA, triiodothyronine (T3) and thyroxine (T4) levels. Additionally, results of partial correlation showed that there was a close relationship between serum Mn levels and the detected indicators accompanied with a positive association between GABA and T3 levels, as well as Gln and T4 levels in the serum of Mn-exposed rats. Unexpectedly, there was no significant correlation between serum Glu and the serum T3 and T4 levels. In conclusion, the results demonstrated that both the Glu/Gln–GABA cycle and thyroid hormone system in the serum may play a potential role in Mn-induced neurotoxicity in rats. Thyroid hormone levels, T3 and T4, have a closer relationship with GABA and Gln levels, respectively, in the serum of rats.

## 1. Introduction

Manganese (Mn), an essential element, acts as a key co-factor in multiple critical enzymatic reactions, including those involved in the metabolisms of lipid, protein, carbohydrate and amino acid neurotransmitter, etc. [1]. The diet, natural presence in the environment, and anthropogenic contaminations of Mn are the main sources of environmental Mn exposure in general populations [2]. Unlike other essential trace elements (e.g., zinc and iron), human dietary Mn deficiency has not been documented [1], and the neurotoxicity of Mn is more prevalent in human populations [3]. Excessive environmental Mn exposure may induce neurotoxicity referred to as manganism [4]. Clinically, manganism is characterized by psychiatric disturbances and an extrapyramidal disorder similar to those observed in Parkinson’s disease (PD) as first described by Couper in 1837. More seriously, an epidemiologic study found that higher Mn concentrations in drinking water are closely related with a lower intelligence quotient in children [5]. However, the exact mechanism of Mn-induced neurotoxicity is unclear.

The alterations of dopamine (DA) and other neurotransmitters in the midbrain have been wildly used as a reliability index to assess the impairment of the central nervous system (CNS) in the animals induced by environmental toxic chemicals. Since manganism shares a similar clinical manifestation with PD, DA alteration has been focused as a main mechanism of Mn-induced neurotoxicity [6,7]. It is noteworthy that the thyroid hormone plays a critical role in regulating the development process of CNS, such as neurotransmitter uptake, synaptogenesis, neuronal plasticity process, etc. [8]. Recently, various heavy metals have been reported to reversely affect the balance of thyroid hormone homeostasis, including iron (Fe), zinc (Zn), arsenic (As), cadmium (Ca), and stibium [8,9,10,11,12]. Elevation of Mn exposure has also been reported to be related with thyroid hormone disorder. For example, an in vivo study indicated that triiodothyronine (T3), thyroxine (T4), and thyroid stimulating hormone (TSH) levels in the serum of Mn-exposed rats were significantly decreased as compared with control [13]. Further, other researchers reported that Mn treatment for 30, 60, and 90 days decreased the T3 and T4 levels in the serum of cocks but induced no changes in serum TSH levels [14]. Additionally, an epidemiology study reported that there was a negative relationship between the elevated Mn levels and thyroid hormone levels in serum [15]. Similarly, studies from other labs found that environmental exposure to high airborne Mn levels induced hypothyroidism in residents via decreasing serum thyroid hormone levels, including T3 and T4 [16].

Interestingly, various studies have shown that there was a closely relationship between glutamate (Glu) and γ-aminobutyric acid (GABA) neurotransmitter cycles and thyroid hormone metabolism [17,18,19]. Glu and GABA, the most predominant excitatory and inhibitory neurotransmitters in the central nervous system (CNS), respectively, play a critical role in maintaining the normal movement performance. It is well known that glutamine (Gln) is the precursor of Glu and GABA. A recent study reported that the Gln/Glu–GABA cycle in CNS is involved in Mn-induced neurotoxicity [20,21,22]. Moreover, an epidemiology study reported that there is a positive correlation between serum GABA levels and the impairment of visual space, behavior, and memory of mild cognitive impairment in PD [23]. Additionally, the alterations of serum Glu and GABA levels have been shown to relate to the neuronal injury in cerebral infarction or stroke patients [24,25]. However, whether excessive Mn exposure may interrupt the balance of Glu/Gln–GABA cycle in serum has not been reported. In this regard, we speculated that there may be a potential role of the Glu/Gln–GABA cycle in thyroid hormone disorders induced by Mn. The present study therefore aimed to explore the effects of sub-acute Mn exposure on the amino acid neurotransmitter system (Glu/Gln–GABA cycle) and thyroid hormone (T3 and T4) in the serum of adult male rats. In addition, the relationship between the serum amino acid neurotransmitter and thyroid hormone levels in Mn-exposed rats was investigated to provide a more comprehensive understanding of the noxious effects of Mn. 

## 2. Methods and Materials

### 2.1. Experimental Animals

Sprague–Dawley (SD) adult male rats (specific pathogen-free grade) were provided by the Experimental Animal Center of Guilin Medical University (SCXKG 2007-0001, Guilin, China). The rats were housed in a room which was maintained at 22 ± 2 °C with a 12/12 h light dark cycle. All procedures performed in this experiment were approved by the Animal Care and Use Committee of Guilin Medical University, China (Ethical approval code: 2018-0002).

### 2.2. Experimental Design

After being fed adaptively for one week, 40 SD adult male rats were selected to perform sub-acute Mn exposure experiment. Rats (185 ± 145 g) were divided randomly into four groups, including control, 7.5, 15, and 30 mg/kg Mn groups, with ten rats in each group. The three doses of Mn-treated groups received intraperitoneal injections of 7.5, 15, and 30 mg/kg MnCl_2_, once per day and five days per week, for four consecutive weeks, while the control group received sterile physiological saline in the meantime. The dosage of Mn was selected based on the published studies [26,27,28]. 

### 2.3. Determination of Mn Levels in Serum

After the experiment, the animals were weighed and anesthetized via receiving intraperitoneal injections of 3.5% chloral hydrate (1 mL/100 g). Blood samples were collected in non-anticoagulant tube from the abdominal aorta. After standing for 30 min at 4 °C, the blood samples were centrifuged (3000 RPM) at 4 °C for 20 min. Serum was then extracted. All serum samples were stored at −80 °C for biochemical measurement. In order to avoid metal contamination, trace element-free materials were used in the present study. Flameless atomic absorption spectrophotometry (Shimadzu AA-6800, Kyoto, Japan) was used to determine Mn levels in the serum as described previously [29]. The instrument setting parameters were listed as followed: wavelength = 279.5 nm; electricity = 10 mA; slit width = 2.0 nm. The serum samples were mixed with matrix modifier with 200 μg/L palladium chloride, 1% nitric acid, and Triton X-100. The mixtures were centrifuged at 4000× *g* for 5 min followed by 20 min standing at room temperature. All samples were performed in triplicate. The accuracy of serum Mn levels was always in the 93%–105% range.

### 2.4. Measurement of Triiodothyronine (T3) and Thyroxin (T4) Levels in Serum

The concentrations of T3 and T4 in the serum were measured by radioimmunoassays using commercial kits (China Institute of Atomic Energy, People’s Republic of China) according to the protocol provided by manufacturers. Radioactivity was determined using an automatic gamma counter. In order to avoid interassay variation, all samples were determined for three times in the same assay. The results were shown as nmol/L for T3 and T4 levels in the serum.

### 2.5. Determination of Amino Acids Neurotransmitters Levels in Serum

The amino acids neurotransmitter (including GABA, Gln, and Glu) levels in the serum were measured using high-performance liquid chromatography (HPLC) analysis based on the previously published articles [26,30]. The serum samples were derived using a derivatization reagent and eluted using the chromatographic condition based on our study [26]. 

Derivatization reagent: 20 μL 2-mercaptoethanol and 4500 μL sodium tetraborate buffer solution (pH = 9.6) were added in 2.7% (w: v) O-phthalaldehyde solutions (dissolved in carbinol) and stored at 4 ℃. The chromatographic condition was listed as follows: 992 mL 50 mmol/L phosphate buffer solution mixed with 8 mL THF (pH = 5.8) was used as the mobile phase “A”, while 750 mL carbinol mixed with 250 mL acetonitrile was used as the mobile phase “B”. The gradient elution was as follows: 0~6 min B%: 25%~25%, 6.01~8 min B%: 25%~42%, 8.01~11 min B%: 42%~50%, 11.01~18 min B%: 50%~75%, 18.01~19 min B%: 75%~95%, 19.01~22 min: B%: 95%~95%, 22.01 min: finished. The detection wavelength: λex = 340 nm, λem = 455 nm. The flow rate: 1 ml/min, the injection volume: 20 μL. The entire chromatography process took 27 min. Results were expressed as μg/μL.

### 2.6. Statistical Analysis

SPSS 13.0 for Windows was used to perform statistical analysis (SPSS Inc, Chicago, IL, USA). Post hoc multiple comparisons were determined using one-way analysis of variance (ANOVA) followed by a Tukey’s test. Partial correlation between amino acid neurotransmitter levels and other indicators were determined using Pearson correlation. When *p*-value < 0.05, the results were considered statistically significant.

## 3. Results

### 3.1. Mn Levels in Serum of Mn-exposed Rats Were Increased

**Figure 1:**
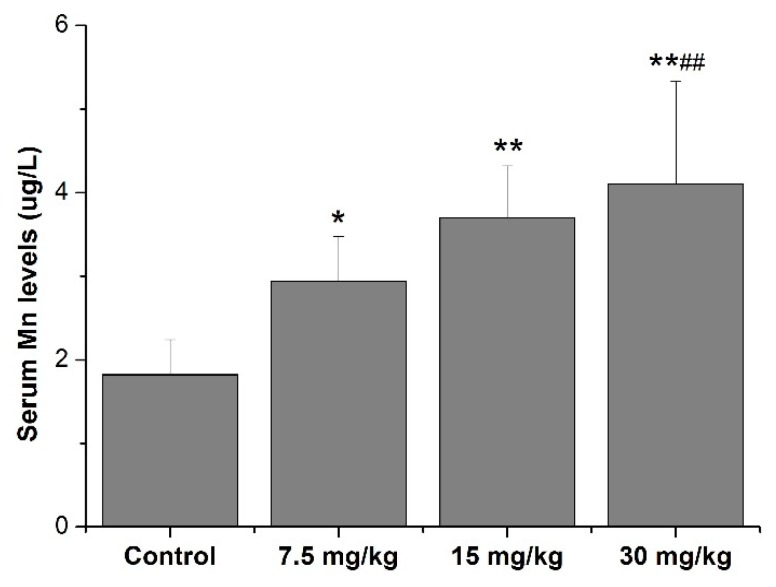
Mn levels in serum of rats. * *p* < 0.05 or ** *p* < 0.01 is marked as statistically significant different from the controls. # *p* < 0.05 or ## *p* < 0.01 is marked as statistically significant different from the 7.5 mg/kg Mn groups. N = 10.

The results in Figure 1 show that Mn levels in the serum of all Mn-treated (including 7.5, 15, and 30 mg/kg Mn) groups were higher than those in the control (*p* < 0.05 or 0.01). Additionally, Mn levels in the serum of the 30 mg/kg Mn group were higher than those in the 7.5 mg/kg Mn group (*p* < 0.01, Figure 1).

### 3.2. Effects of Mn Treatment on T4 and T3 Levels in Serum of Rats

**Figure 2:**
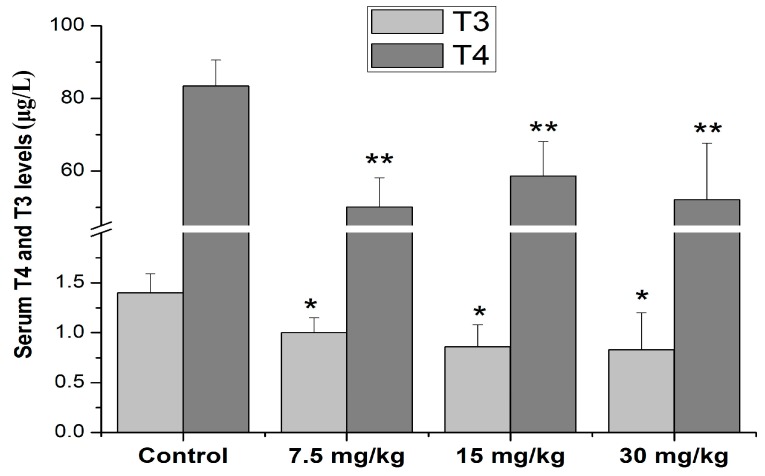
Effects of Mn on serum T3 and T4 levels in male rats. * *p* < 0.05 or ** *p* < 0.01 is marked as statistically significant different from the controls. N = 10.

Compared with the control, the serum T4 levels in all Mn-exposed group doses were decreased (50.1 ± 8.1 μg/L, 58.7 ± 9.5 μg/L, 52.2 ± 15.5 μg/L in 7.5 mg/kg, 15 mg/kg, and 30 mg/kg Mn groups, respectively, vs. 83.4 ± 7.2 μg/L in the control group, *p* < 0.01). Moreover, the results also showed that serum T3 levels in the 7.5, 15, and 30 mg/kg Mn groups were lower than those of the control (0.996 ± 0.19, 0.86 ± 0.27, and 0.83 ± 0.37 μg/L in different Mn groups, respectively, vs.1.40 ± 0.19 μg/L in the control group, *p* < 0.05, Figure 2).

### 3.3. Effects of Mn Treatment on Serum Amino Acid Neurotransmitter Levels of Rats

**Figure 3:**
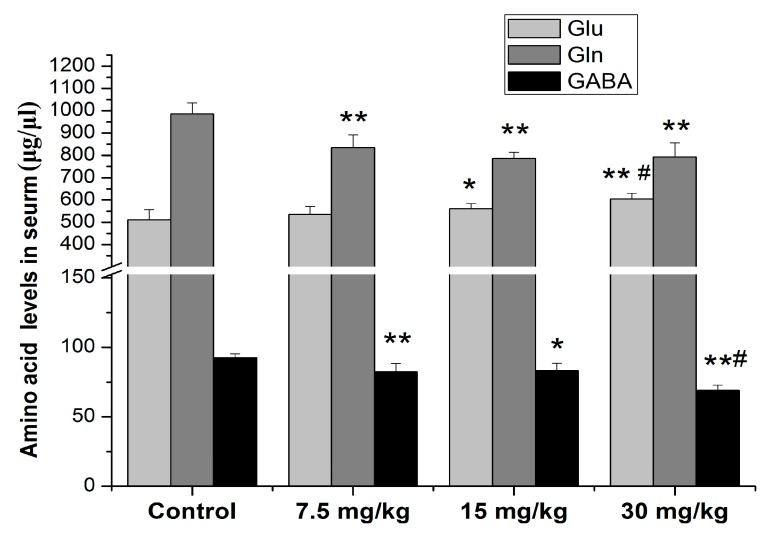
Effects of Mn treatment on serum neurotransmitter levels in male rats. * *p* < 0.05 or ** *p* < 0.01 is marked as statistically significant different from the controls; # *p* < 0.05 is marked as statistically significant different from the 7.5 mg/kg Mn group. N = 10.

A similar change trend was shown on serum Gln and GABA levels after Mn exposure for 4 weeks (Figure 3). When the treatment concentration of MnCl_2_ reached 15 mg/kg, the serum Glu levels of the 15 and 30 mg/kg Mn groups were higher than those in the control (*p* < 0.05 or 0.01). Further, the serum Glu levels of the 30 mg/kg Mn group were higher than those in the 7.5 mg/kg Mn group (*p* < 0.05), but not in the 15 mg/kg Mn group (*p* > 0.05). Additionally, the serum Gln and GABA levels of the 7.5, 15 and 30 mg/kg Mn groups were decreased as compared to the control (*p* < 0.05 or 0.01). The serum GABA levels of the 30 mg/kg Mn group were lower than those in the 7.5 mg/kg Mn group (*p* < 0.05, Figure 3). 

### 3.4. Partial Correlation between Serum Mn Levels and Other Indicators

The relationships between serum Mn levels and other indicators are shown in Table 1. The serum GABA concentrations were positively associated with serum Gln and T3 concentrations (r = 0.652 or 0.423, *p* < 0.01 or 0.05), but negatively associated with the serum Mn and Glu concentrations (r = −0.782 or −0.601, *p* < 0.01). Moreover, there was a significant negative correlation between Gln and Mn, Glu concentrations in the serum of rats (r = −0.679 or −0.484, *p* < 0.01). By contrast, serum Gln concentrations were in a positive association with serum T4 concentrations (r = 0.490, *p* < 0.01). Additionally, a negative association was also found between Mn concentrations and T3 and T4 concentrations in the serum (r = −0.618 or −0.719, *p* < 0.01). However, there was no significant correlation between serum Glu and serum T3 and T4 concentrations (r = −0.225 or 0.393, *p* > 0.05).

## 4. Discussion

Our present results clearly showed that sub-acute Mn exposure may interrupt the balance of the amino acid neurotransmitter, and thyroid hormones in the serum of rats via changing the levels of Glu, Gln, GABA, T3, and T4 in the serum. Furthermore, we also found a closely relationship between serum Mn levels and the abovementioned indicators. However, we only found a positive correlation between GABA and T3, and Gln and T4 concentrations in the serum of Mn-exposed rats.

Mn, an essential metal in human body, is found widely in the environment, while excessive Mn exposure contains a potent neurotoxic effect on the central nervous system. In this regard, a useful biomarker for detecting the Mn exposure status is the prerequisite for early assessment of the onset of manganism [31,32,33]. Blood Mn levels (including whole blood, erythrocytes, plasma, serum) have been widely used as a biomarker to determine environmental or occupational Mn exposure, because they are easily obtained biomarkers and have no external contamination factors [34]. Nevertheless, investigators have pointed out that there are some drawbacks to using the whole blood Mn levels as a biomarker for the assessment of the status of Mn exposure. Firstly, a toxic kinetics study demonstrated that whole blood Mn levels have a very short t1/2 of only 2 hours [35] while the t_1/2_ is about 20–40 days in human body [36], suggesting it is inconsistent with the actual Mn levels in tissues. Secondly, whole blood Mn levels contain various variations among the individuals [37]. For these reasons, some researchers considered that serum Mn levels seem to be more accurate and reliable as a biomarker to detect the Mn exposure status for it is sensitive to various variations in dietary intake of Mn [38]. Moreover, serum Mn levels have a similar t_1/2_ with Mn levels in the tissues ranging between 13 and 37 days, suggesting serum Mn levels and tissues Mn levels seem to be concordant after short-term Mn exposure [39]. Additionally, Mirmohammadi et al. [40] demonstrated that there is a straight relationship between indoor air Mn levels and serum Mn levels of the occupational Mn-exposed workers. Similarly, some studies reported that serum Mn levels were closely related with the neurological impairment of occupational workers [41] and attention deficit hyperactivity disorder in children. Herein, the present study determined the serum Mn levels and showed that serum Mn levels of Mn-treated rats were increased with a correlation coefficient of 0.733 (Figure 1). Additionally, the results also showed that serum Mn levels are associated with the amino acid neurotransmitter and thyroid hormone levels in the serum of Mn-exposed rats (Table 1).

The thyroid hormone plays a critical role in maintaining cellular metabolism and normal CNS development [42]. Currently, serum T3 and its precursor T4 levels have been commonly used as indicators for the assessment of thyroid function. A series of studies have demonstrated that low serum T3 and T4 levels may cause reverse effects on the normal development of CNS via inducing synaptic plasticity impairment and decreasing neuronal cells in the hippocampus [43,44]. An epidemiological study reported that a deficient of thyroid hormones produced may be induced by environmental toxicants and closely correlated with brain development impairment in children characterized with cognitive deficits and mental retardation [45,46]. Furthermore, low serum T4 and T3 levels have also been shown to be correlated with reproductive disorders [47]. It is worth noting that excessive metals exposure may adversely affect normal CNS development and the reproductive system via destroying the balance of thyroid hormones system, including Mn [15,48]. For example, an early study showed that Mn chloride (10 and 20 mg/kg) treatment for 24 hours markedly decreased thyroid hormone levels in serum, the percentages of iodinated thyronines, and the protein-bound iodide fraction in the thyroids of rats [49], which was confirmed by a subsequent study [50]. Furthermore, Hoseini et al. [51] showed that Mn treatment increased the serum T3 and T4 levels, but has no effects on the thyroid T3 and T4 levels. Additionally, an epidemiologic study on occupational Mn-exposed workers demonstrated that excessive Mn exposure caused an imbalance in the thyroid hormones via decreasing the serum thyroid-stimulating hormone (TSH), testosterone (TST), prolactin (PRL), and follicle-stimulating hormone (FSH) concentrations [15]. The present study showed that Mn chloride treatment for 4 weeks interfered on the balance of thyroid hormones, as indicated by a decrease in the serum T4 and T3 levels, although the serum T3 and T4 levels showed a similar pattern in the present study, as T3 and T4 were decreased in all treatment groups (Figure 2). Changes in the serum T4 levels were more obvious than changes in the serum T3, suggesting that excessive Mn exposure may have a potential effect on monodeiodinase activity, which is responsible for the conversion of T4 into T3 and needs to be further studied. These contradictory results may result from the different treatment routes, Mn compound, doses, and species which were used in these studies. 

GABA and Glu, the main neurotransmitter amino acids, are known to play a key role in maintaining normal learning and movement abilities [52,53]. Thus, impairments of the Gln/Glu–GABA cycle in the brain are associated with movement disorders and motor deficits, and they are involved in the genesis and development of neurodegenerative disease, such as idiopathic PD, Huntington’s disease, amyotrophic lateral sclerosis (ALS), etc. [54,55,56,57]. It is well known that injury of dopaminergic neurons is involved in neurotoxicity induced by Mn [4,56]. Interestingly, the interactions between DA and GABA neurons play critical roles in regulating dopaminergic neurons. The stimulation of GABA inputs from multiple brain regions to midbrain dopaminergic neurons via activating the GABA receptor may inhibit the firing of these neurons, suggesting that excessive Mn exposure may also interfere with the metabolism of GABA [2,9]. Recently, many studies have demonstrated that there is a correlation between brain Mn levels and impairment of the Glu/Gln–GABA cycle. However, the results of these studies are contradictory. For example, Lipe et al. [58] showed that sub-acute Mn exposure increased the Glu, Gln, and GABA levels in the cerebellum of adult rats, but only decreased Gln levels in the hippocampus and caudate nucleus of weanling rats. Recently, an epidemiologic study showed that the elevated thalamic GABA levels were associated with the Mn exposure levels of occupational Mn-exposed workers [59]. By contrast, other studies demonstrated that excessive Mn exposure caused a decrease in brain GABA, olfactory bulb Gln, and GABA levels [26,60], while studies from other labs showed that there were no changes in these neurotransmitters levels in the brain of Mn-exposed rats [61]. The present study firstly determined the effects of Mn on the Glu/Gln–GABA cycle in the serum of rats. The results showed that all treatment groups showed a decrease in the serum Gln and GABA levels of rats, while serum Glu levels slightly increased in the 15 and 30 mg/kg Mn groups compared to the control, suggesting that the serum Gln and GABA levels were more sensitive to Mn exposure than serum Glu levels (Figure 3). Moreover, the results also showed that serum Mn levels were negative correlated with serum Gln and GABA levels and positive correlated with serum Glu levels (Table 1). These findings suggested that sub-acute Mn exposure adversely affects serum amino acids neurotransmitters. The slight alterations in serum Glu would be explained by the conversion of Gln into Glu, and Glu into GABA by phosphate activated glutaminase and Glu decarboxylase, respectively [13].

Soldin and Aschner concluded that Mn may indirectly affect thyroid hormone homeostasis via dopamine dysregulation [62]. Interestingly, Wiens and Trudeau reported that thyroid hormone levels have a strong negative effect on the development of the GABA system [18]. Further, GABA is effective against different symptoms induced by deficient thyroid hormone production, such as memory impairment, fatigue, and depression [19,63]. Moreover, an in vitro study demonstrated that GABA treatment increased the synthesis of T3 and T4 and the ratio of T3/T4 in thyroid follicular epithelial cells [64]. Additionally, an in vivo study also showed that GABA has protective effects against the decrease of T3 and T4 levels in the blood of mice induced by fluoride [17]. It is noteworthy that T3 affected the Glu levels via increasing mRNA and protein expressions of the Glu transporters, including Glu aspartate transporter, glial Glu transporter-1, etc. [65]. The aforementioned data suggest that the Glu/Gln–GABA cycle plays a potential role in modulating the balance of the thyroid hormone. To date, none of these studies have investigated the relationship between the alterations of the Glu/Gln–GABA cycle and thyroid hormone induced by Mn. The results of the present study showed that GABA levels were negatively correlated with Glu levels (r = −0.601) but positively correlated with Gln and T3 concentrations in serum (r = 0.652 or 0.423). Further, a positive correlation was also found between Gln and T4 levels in the serum of Mn-exposed rats. However, we did not find any relationship between Glu concentrations and T3 and T4 levels in the serum of rats (Table 1). These results indicate that GABA and Gln may play a potential role in Mn-induced thyroid hormone disorder.

## 5. Conclusions

In conclusion, MnCl_2_ injection elevated Mn and Glu levels, inhibited GABA, Gln, and thyroid hormones’ T3 and T4 levels in the serum of rats. Serum Mn levels correlate with Glu, Gln, GABA, ans thyroid hormones’ T3 and T4 levels in the serum. The results certified the hypothesis that both the Glu/Gln–GABA cycle and thyroid hormone play a potential role in Mn-induced neurotoxicity in rats. Moreover, the results of partial correlation analysis suggest that there is a positive association between serum GABA and T3, as well as Gln and T4 levels. Unexpectedly, there was no significant correlation between serum Glu and the serum T3 and T4 levels. However, the exact mechanism for the modulatory effects of the Glu/Gln–GABA cycle on thyroid hormones induced by Mn are not yet well understood. Thus, this mechanism needs to be further studied.

## Figures and Tables

**Table 1:** Partial correlation between serum Mn levels and other indicators.

Indicator	Mn	Glu	Gln	GABA	T3
Mn	-	-	-	-	-
Glu	0.756 **	-	−0.484 **	−0.601 **	-
Gln	−0.679 **	-	-	0.652 **	-
GABA	−0.782 **	-	-	-	-
T3	−0.618 **	0.225	−0.120	0.423 *	-
T4	−0.719 **	0.393	0.490 *	0.274	−0.322

Note: * *p* < 0.05, ** *p* < 0.01.
